# Evaluating Parameter Value Identification Methods for Modeling of Nonlinear Stress Relaxation in Polyethylene

**DOI:** 10.3390/ma18132960

**Published:** 2025-06-23

**Authors:** Furui Shi, P.-Y. Ben Jar

**Affiliations:** 1 College of Mechanical and Automotive Engineering, Ningbo University of Technology, Ningbo 315336, China; 2 Department of Mechanical Engineering, University of Alberta, 10-203 Donadeo Innovation Centre for Engineering, 9211-116 Street NW, Edmonton, AB T6G 1H9, Canada; ben.jar@ualberta.ca

**Keywords:** relaxation, modeling, mechanical properties, polyethylene

## Abstract

Viscous properties play a major role in the time-dependent deformation behavior of polymers and have long been characterized using spring-dashpot models. However, such models face a bottleneck of having multiple sets of model parameter values that can all be used to simulate the same deformation behavior. As a result, these model parameters have not been widely used to quantify the viscous properties. In this study, a newly developed multi-relaxation-recovery test was used to obtain the variation in stress response to deformation of polyethylene (PE) and its pipes during relaxation, revealing the complexity of PE’s nonlinear viscous stress response to deformation. Using a three-branch spring-dashpot model with two Eyring’s dashpots, this study shows the possibility of determining the model parameter values using four different analysis methods, namely, the mode method, peak-point method, highest-frequency method, and best-five-fits method. Model parameter values from these methods are compared and discussed in this paper, to reach the conclusion that the best-five-fits method provides the most reliable and relatively unique set of model parameter values for characterizing the mechanical performance of PE and its pipes. The best-five-fits method is expected to enable the use of the model parameters to quantify PE’s viscous properties so that PE’s load-carrying performance can be properly characterized, even for long-term applications.

## 1. Introduction

It is widely accepted that the ages of human civilizations are defined by their utilization of materials. For instance, people in the Stone Age learned how to use stones and advanced the development of society. We are currently living in the Polymer Age [1,2]. Nowadays, the majority of global polymer production is in semi-crystalline polymers (SCPs) [3]. SCPs, such as polyethylene (PE), are materials made of large molecules and belong to a class of thermoplastics with a complicated microstructure, consisting of both crystalline and amorphous phases [4,5]. The study of SCPs has attracted enormous attention throughout the world, and the number of publications about SCPs has steadily increased in recent years [6]. It was reported that more than 98% of plastic pipes were made of PE in 2021 [7], which suggests that PE is widely used for gas pipe applications in the U.S. [8,9]. However, PE exhibits a nonlinear time-dependent behavior under loading in the form of stress relaxation, which was believed to originate from the sliding between long molecular chains in amorphous regions [10]. Stress relaxation behavior can lead to the leakage of PE pipes, which can result in the failure of the pipe transportation system [11,12,13,14,15]. A winner of the Nobel Prize in Physics, Richard Feynman, clearly showed how the time-dependent behavior of polymers is significant in practical engineering after the explosion of the space shuttle Challenger caused by the failure of an O-ring in 1986 [16]. Therefore, it is necessary to fully characterize the relaxation behavior of PE [8].

Half a century ago, people proposed a stress relaxation test for plastic pipes under constant strain in order to determine their material properties [17,18]. Zhang and Moore [19] investigated the relaxation behavior of high-density polyethylene (HDPE) using different loading rates and deformation levels under uniaxial compression and used the experimental data to develop the viscoplastic (VP) constitutive model. It was found that the VP model can provide good simulation of the relaxation data from the experiments. However, the maximum difference in stress between the model and experiments was almost 1 MPa. In addition, the period of the relaxation behavior in the experiments did not exceed 4000 s. Hong and Strobl [20] conducted relaxation tests on PE at different deformation levels and simulated the relaxation behavior using a three-branch model based on Eyring’s law, but they assumed the characteristic relaxation time to be a constant. Drozdov and Christiansen [21] found that the dimensionless stress–time curves are practically independent of deformation from the relaxation test results for PE at different strains, and they derived a constitutive model by fitting the observed data in the experiments. However, they only considered the relaxation tests below the yield point. Recently, Zhang and Jar [22] adopted a two-branch spring-dashpot model with an Eyring’s dashpot and determined the quasi-static stress–strain relationship of PE pipes by removing the viscous stress of the relaxation behavior at different strains. Tan and Jar [23] developed the multiple-relaxation (MR) test and identified the critical deformation level for the onset of the plastic deformation in the crystalline phase using the standard model. Then, we [24] observed the unusual stress response of the recovery behavior in a novel multiple-relaxation-recovery (RR) test on PE and constructed a three-branch model with two Eyring’s dashpots for the simulation of unusual stress response. However, the question of how unique the estimated parameter values are remains open for the three-branch model [10,25,26]. It is necessary to develop a parameter value identification method to obtain unique parameter values. Such a method is the subject of this paper.

This paper introduces the mode method, peak-point method, highest-frequency method, and best-five-fits method for the identification of the model parameter values of a three-branch model on the relaxation behavior of HDPE cylindrical specimens and PE-Xa NPR specimens. RR tests on HDPE cylindrical specimens and PE-Xa NPR specimens are performed. The fitting equation from the three-branch model is derived for the simulation of the relaxation behavior. The algorithms for the simulation using the fitting equation are described for the four presented methods. Subsequently, the model parameter values are determined and compared. It is demonstrated that the best-five-fits method provides the most reliable and relatively unique set of model parameter values for both HDPE cylindrical specimens and PE-Xa NPR specimens.

## 2. Experiments

The RR tests were conducted at room temperature using the Qualitest Quasar 100 universal test machine (Qualitest, Lauderdale, FL, USA), and the test data were collected using a personal desktop [23]. The stroke as a function of time for the RR test was presented in a previous work [24,27]. The RR test consists of six stages in one cycle: 1st loading, relaxation, 2nd loading, stabilization (at a constant stroke), unloading, and recovery stages. The maximum deformation introduced in the RR tests was set to exceed the yield point, at which approximately 30 cycles were generated [23]. The crosshead speed was set to be 1 mm/min, with 10,000 s allocated for each relaxation, stabilization, or recovery stage.

**Figure 1:**
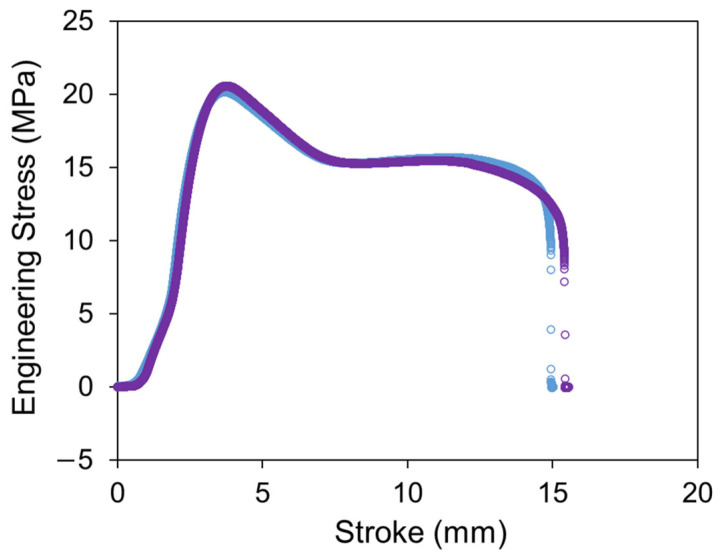
Engineering stress versus stroke of uniaxial tensile tests on two PE-Xa NPR specimens.

Two types of specimens were used to conduct the RR tests: an HDPE cylindrical specimen and PE-Xa notched-pipe-ring (NPR)specimen. The HDPE cylindrical specimen is HDPE-b in the literature [24]. The characteristics of the PE-Xa NPR specimen can be found in our previous work [28]. The results of uniaxial tensile tests on two PE-Xa NPR specimens, conducted with a Qualitest Quasar 100 universal test machine, are shown in Figure 1. From Figure 1, the yield stress is between 20 MPa and 21 MPa, and the stroke for the yield point is between 3.5 mm and 4 mm.

**Figure 2:**
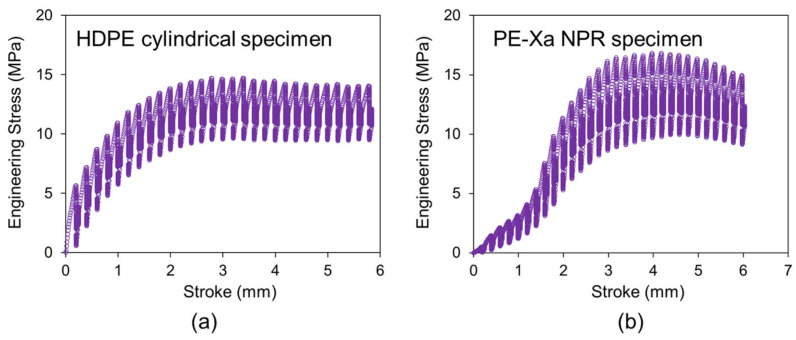
Engineering stress versus stroke in RR test on (**a**) HDPE cylindrical specimen and (**b**) PE-Xa NPR specimen.

To ensure repeatability and reliability, two RR tests were conducted for each material. Figure 2a and Figure 2b show engineering stress as a function of stroke for the RR test on an HDPE cylindrical specimen and PE-Xa NPR specimen, respectively. It is observed that the maximum engineering stress of the PE-Xa NPR specimen is higher than that of the HDPE cylindrical specimen.

## 3. Three-Branch Model

**Figure 3:**
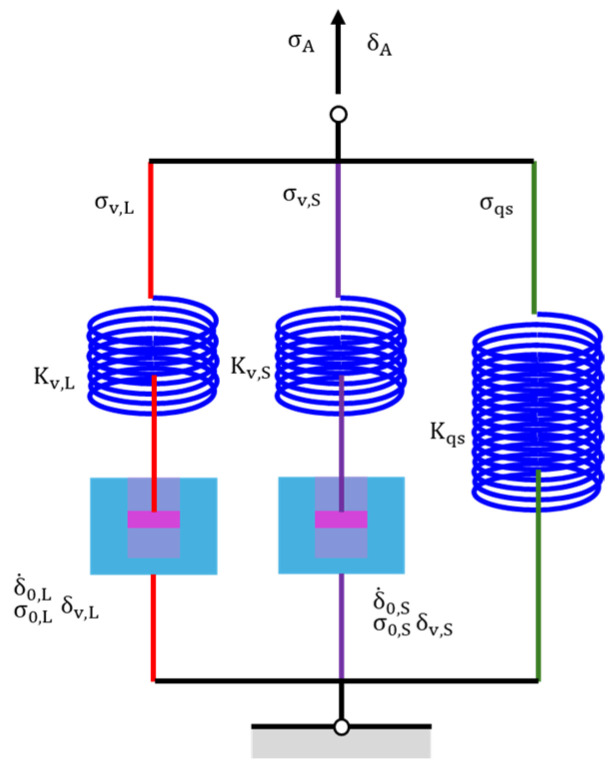
The three-branch spring-dashpot model adopted in this paper.

It is widely accepted that the one-branch model is insufficient to describe the relaxation behavior of PE, as the applied stress in PE consists of both time-dependent and time-independent components [20]. Tan and Jar [23] found that the relaxation behavior of PE can be described by a two-branch spring-dashpot model with a single dashpot. Although the long-term relaxation behavior can be mimicked well, the accurate simulation of the short-term relaxation behavior was missed. Our previous work [24] found that the two-branch spring-dashpot model, with two dashpots connected either in series or in parallel, failed to simulate the unusual stress response observed during the recovery behavior of HDPE. Recently, it was reported that the three-branch model can accurately simulate the relaxation and recovery behavior of HDPE [27]. In this paper, the three-branch model is adopted to analyze the stress relaxation from the RR tests, which is shown in Figure 3. The three-branch model contains long-term viscous branch, short-term viscous branch, and quasi-static branch, which are denoted using subscripts *L*, *S*, and *qs*. In our previous work [27], it was found that the long-term viscous branch is capable of capturing the time-dependent, long-term viscous stress (the component in PE with a longer characteristic relaxation time). The short-term viscous branch is capable of capturing the time-dependent, short-term viscous stress (the component in PE with a shorter characteristic relaxation time). The quasi-static branch is capable of capturing the time-independent stress (the time-independent component in PE). The prediction ability of the model was examined in our previous work [27], and the results indicate that the values from the prediction of long-term viscous stress in the relaxation stage show a consistent trend of variation with long-term viscous stress calculated in the following recovery stage and a study to evaluate the model’s performance under different deformation scenarios and further assess its predictive capability when this manuscript was prepared. This model and its governing equations can also be found in our previous work [27]. Therefore, the stress change during each relaxation (recovery) stage can be written as follows:

(1) $$\begin{array}{l} {∆\sigma }_{A}=\sigma_{A}(0)-\sigma_{A}(t)\\ =\sigma_{v,L}(0)+\sigma_{v,S}(0)\\ -2\sigma_{0,L}{tanh}^{-1}\{tanh[\sigma_{v,L}(0)/({2\sigma }_{0,L})]\exp(-t/\tau_{v,L})\}\\ -2\sigma_{0,S}{tanh}^{-1}\{tanh[\sigma_{v,S}(0)/\left({2\sigma }_{0,S}\right)]\exp\left(-t/\tau_{v,S}\right)\} \end{array}$$

with

(2) $$\tau_{v,i}=\sigma_{0,i}/(K_{v,i}{\dot{\delta }}_{0,i})$$

where $\sigma_{A}$ represents the applied engineering stress, t the time calculated from the beginning of each stage, $\sigma_{A}(0)$ the applied engineering stress at t=0, $\sigma_{v,i}(0)$ the viscous stress at t=0, $\sigma_{0,i}$ reference stress, $\tau_{v,i}$ characteristic relaxation time, $K_{v,i}$ the spring stiffness, and ${\dot{\delta }}_{0,i}$ the reference stroke rate, with i = L or S.

## 4. Parameter Value Identification Methods

For the simulation of nonlinear stress relaxation, multiple solutions exist for the model parameter values of the three-branch model. In the previous work, 10 sets of model parameter values of HDPE for the PE100 pipe were determined using the mode method [27]. Then, the best-five-fits method was proposed, which was applied to the determination of the model parameter values of HDPE, PE-Xa pipe, PE4710-yellow pipe, PE4710-black pipe, and PE2708 pipe in the previous work [28]. In this paper, the novel peak-point method and highest-frequency method were proposed. Therefore, the four methods are summarized in this section and were used to analyze the data for the comparison in this paper. In addition, the programming of the four methods was developed using MATLAB 2024a.

### 4.1. Mode Method

**Figure 4:**
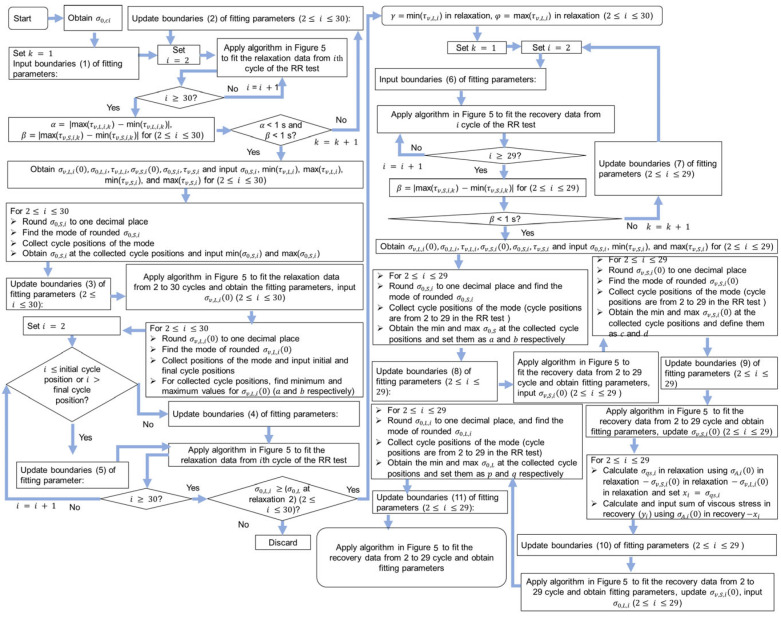
Flow chart of the mode method for model parameter value identification.

The details of the mode method for the determination of the model parameter values in the relaxation and recovery stages of RR tests are shown in Figure 4, which were also illustrated in the previous work [27]. Equation (1) was used to fit the relaxation data and recovery data, and all the fitting parameter values in the relaxation and recovery stages were obtained. Because 28 cycles of the RR test for the NPR specimen were collected, 28 was used in the flow chart in Figure 4. For the determination of the fitting parameters in the relaxation stages in Figure 4, the ranges of $\sigma_{0,L}$ values were first obtained. It is important to note that the ranges of $\tau_{v,L}$ and $\tau_{v,S}$ values were narrowed down until their variation for the entire RR test was less than 1 s. The ranges of $\tau_{v,L}$ and $\tau_{v,S}$ were determined using their averages in the 28 relaxation stages. The ranges and plateau for $\sigma_{0,S}$ and $\sigma_{v,L}(0)$ were calculated using their modes. A plateau region from around 3 mm to 6 mm in Figure 2a was observed, and the plateau region of model parameter values during deformation was also reported in the literature [23,24]. Therefore, $\sigma_{v,L}(0)$ values were assumed to show the plateau region during deformation. In addition, quasi-static stress ($\sigma_{qs}$) values were assumed to be the same for the relaxation and the recovery stages at the same deformation level, which is consistent with the finding reported in the literature [29,30]. It should be noted that the mode for this method represents the mathematical concept, that is, the most common number that appears in one set of numbers. The initial boundaries for the six fitting parameters in the relaxation stages are [0.1, 20] (in MPa) for $\sigma_{v,L}(0)$, [0.01, 2] (in MPa) for $\sigma_{0,L}$, [1000, 90,000] (in s) for $\tau_{v,L}$, [0.1, 20] (in MPa) for $\sigma_{v,S}(0)$, [0.01, 2] (in MPa) for $\sigma_{0,S}$, and [1, 900] (in s) for $\tau_{v,S}$. From Figure 4, index i in the flow chart is used to represent the cycle number for the RR test so that the fitting parameter values of each cycle of the RR test can be determined. Values for reference stress ($\sigma_{0,ci}$), with subscript ci denoting the cycle i of the RR test, were determined using the curve fitting method proposed in the literature [23]. That is, $\sigma_{0,ci}$ values were calculated by fitting the short-term behavior of the relaxation stages using a standard model with a single Eyring’s dashpot. Notably, the terms ‘max’, ‘min’, and ‘avg’ represent operations for determining the maximum, minimum, and average values within an array, respectively. The ranges of the boundaries used in Figure 4 are shown in the Supplementary Materials.

**Figure 5:**
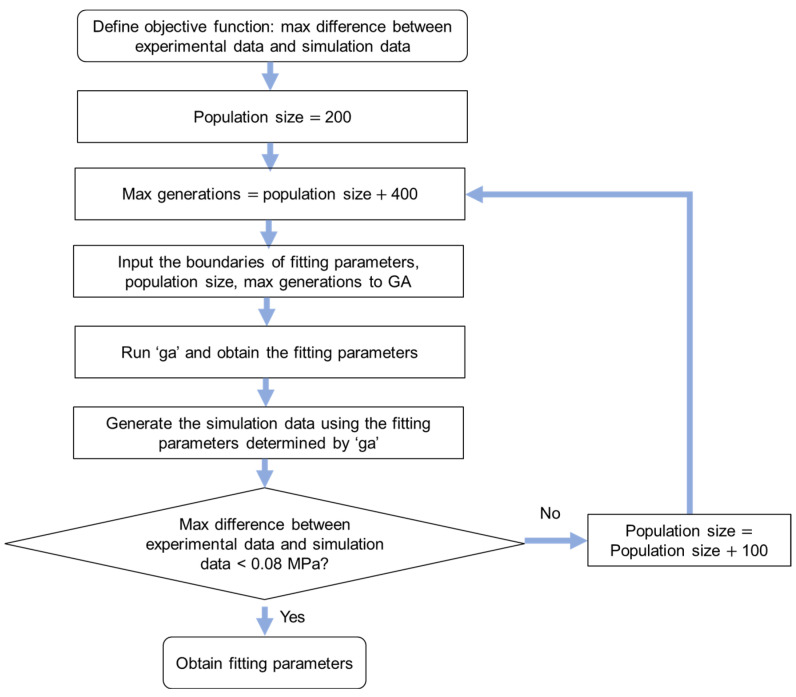
Model parameter value searching method based on ‘ga’ in MATLAB [27].

A genetic algorithm was used to search for the optimal model parameter values and is shown in Figure 5. Function ‘ga’ in MATLAB was used to implement the genetic algorithm. The upper boundary of the population size is 1500, hence the max difference between experimental data and simulation data was checked after running the algorithm shown in Figure 5. The programming in Figure 4 can be terminated and restarted when the max difference between experimental data and simulation data is larger than 0.08 MPa. Therefore, the max difference between experimental data and simulation data from results using the mode method is less than 0.08 MPa, and this value is much smaller than the values reported in the literature, such as 0.17 MPa [30], 0.3 MPa [31], 0.4 MPa [23], and 1 MPa [32].

### 4.2. Peak-Point Method

**Figure 6:**
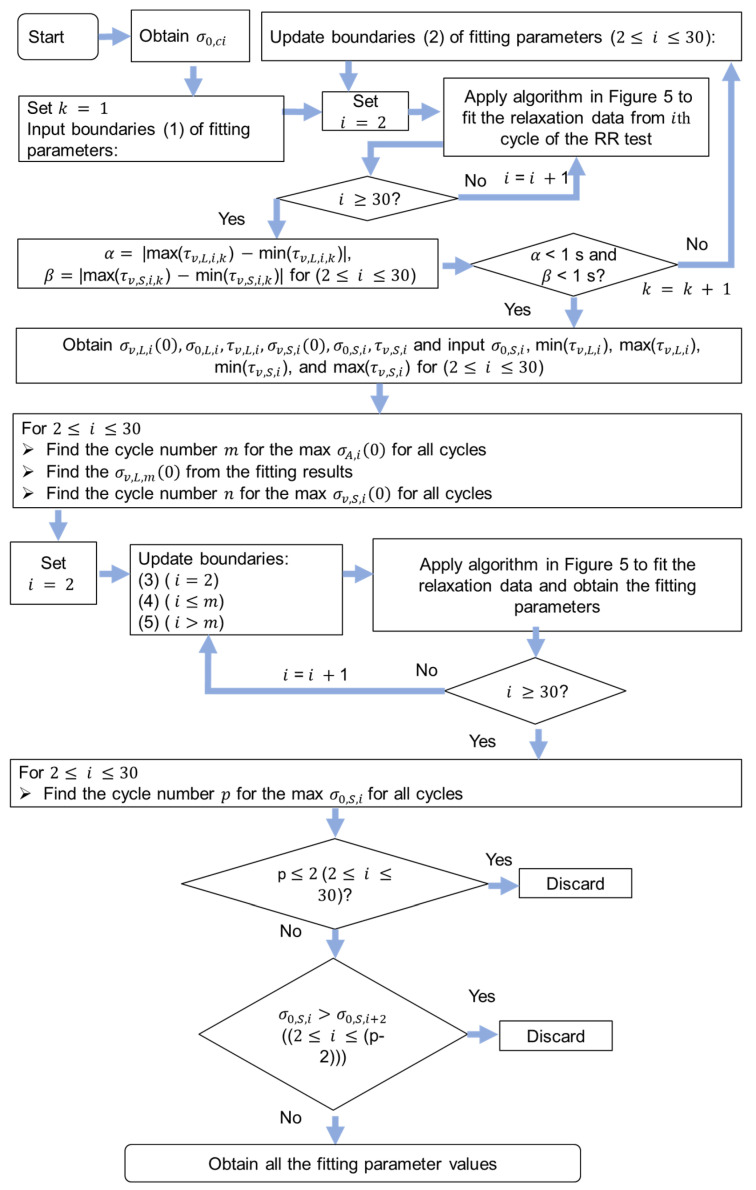
Flow chart of the peak-point method for parameter value identification.

From Figure 2b, the experimental data of the NPR specimen show no plateau region but only a peak; hence, a novel peak-point method was developed for the determination of the model parameter values of NPR specimens. The flow chart in Figure 6 depicts the programming of the peak-point method for the parameter value identification in the relaxation stages of the RR tests. Similarly, Equation (1) was used to fit the relaxation data, and all the fitting parameter values in the relaxation stages were obtained. $\sigma_{0,ci}$ values were calculated using the same method from the mode method. From Figure 6, the ranges of $\sigma_{v,S}\left(0\right)$ and $\sigma_{v,L}\left(0\right)$ values were determined using their peak points. The peak point $\sigma_{v,L}\left(0\right)$ was assumed to be at the same stroke of the peak point of $\sigma_{A}\left(0\right)$. Because 30 cycles of the RR test for the NPR specimen were collected, 30 was used in the flow chart in Figure 6. The algorithm in Figure 5 was also used to search for the optimal fitting parameter values, but value 0.08 in Figure 5 was changed to be 0.1. Therefore, the maximum difference between experimental data and simulation data from the results using the peak-point method was less than 0.1 MPa. The ranges of the boundaries used in Figure 6 are shown in the Supplementary Materials.

Although the mode method and peak-point method can be used to determine the model parameter values for the cylindrical specimens and NPR specimens, they consist of several assumptions, such as the assumptions for the determination of $\sigma_{0,ci}$ and the assumption of constant $\tau_{v,L}$ and $\tau_{v,S}$ during deformation. To remove these assumptions, the highest-frequency method and best-five-fits method were developed.

### 4.3. Highest-Frequency Method

**Figure 7:**
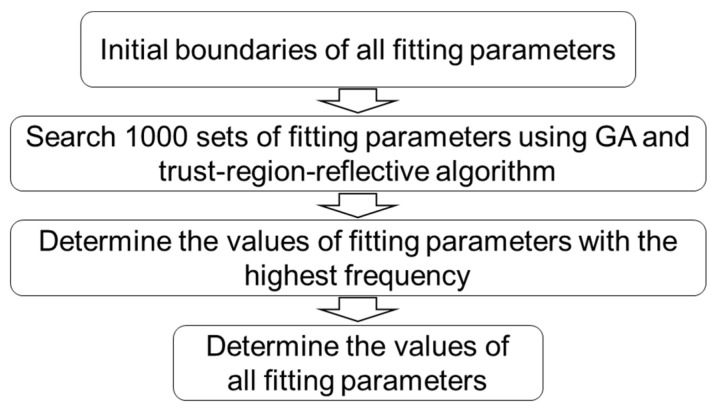
Procedures of the highest-frequency method for parameter value identification.

**Figure 8:**
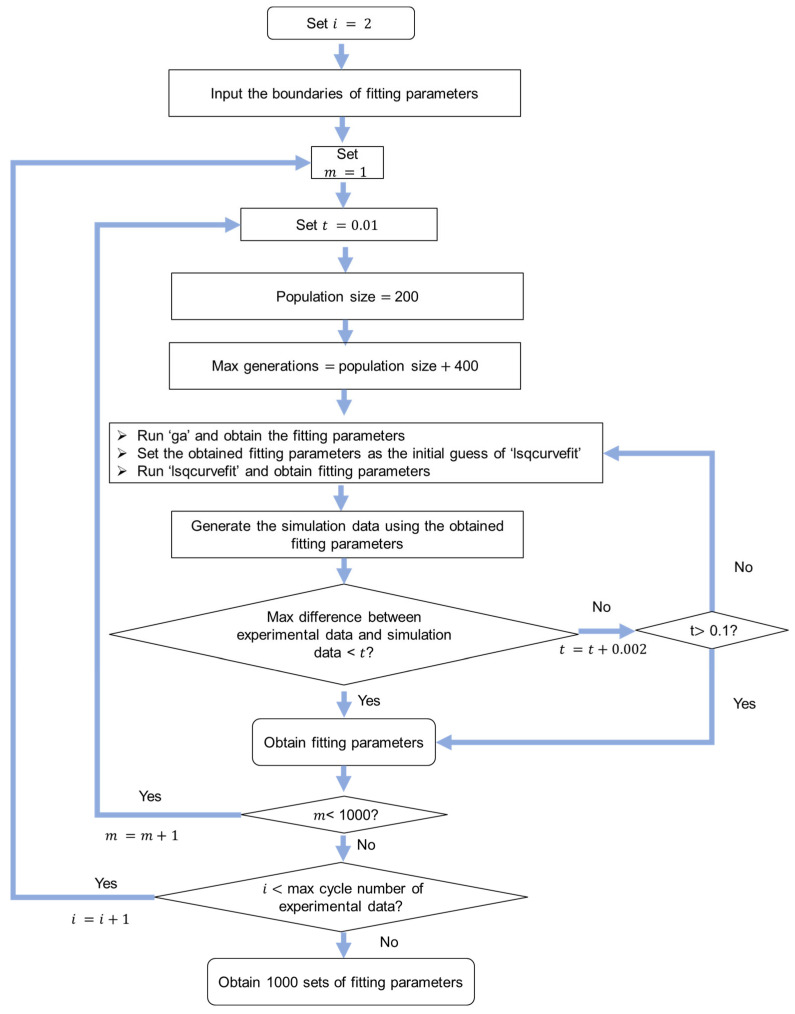
Procedure to search for the 1000 sets of model parameter values.

**Figure 9:**
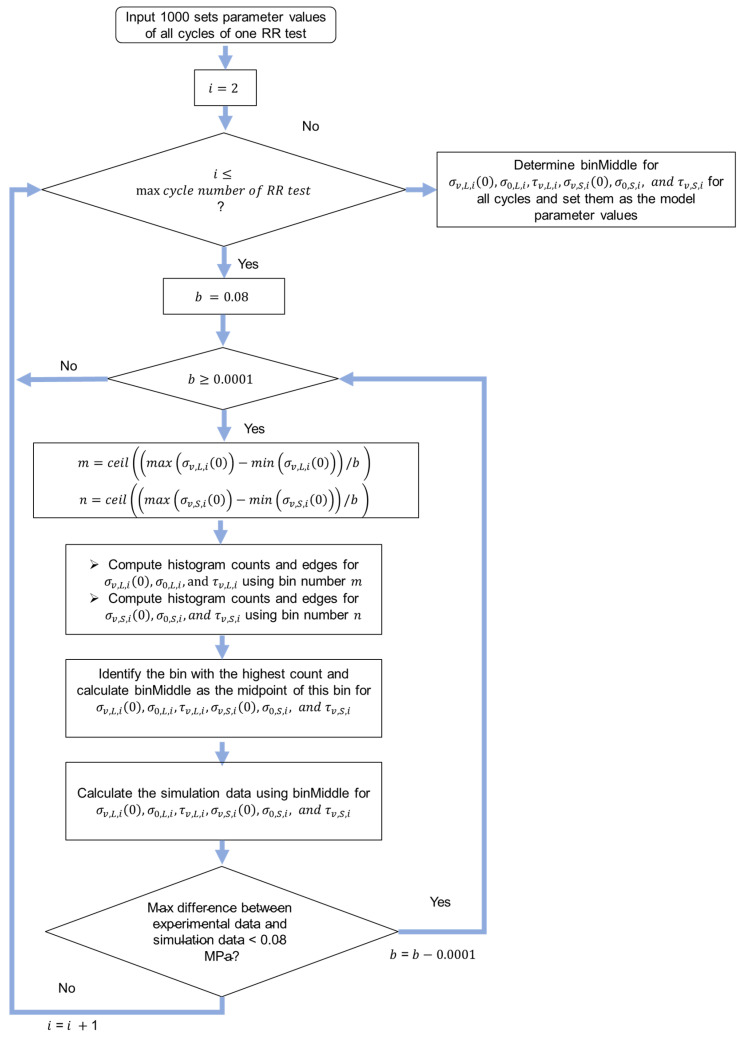
Procedure to determine the model parameter values with the highest frequency.

The flow chart in Figure 7 shows the algorithm for the highest-frequency method. Similarly, Equation (1) was used for the curve fitting of experimental data of relaxation stages. The initial boundaries for fitting parameter were set to be the same as those in the previous two methods in Section 4.2 and Section 4.3. Function ‘parfor’ was used for the parallel computing to speed up the process to obtain the 1000 sets of model parameters. Functions ‘ga’ and ‘lsqnonlin’ in MATLAB were used to search for the optimal fitting parameters. Function ‘lsqnonlin’ was based on the trust-region-reflective algorithm. The algorithm in Figure 8 was used to search for the 1000 sets of model parameter values. Figure 9 shows the procedure to determine the model parameter values with the highest frequency. Finally, the model parameter values that could generate a maximum difference between experimental data and simulation data of less than 0.08 MPa were selected. Function‘ceil’ in MATLAB was used to determine the nearest integer greater than or equal to a number.

### 4.4. Best-Five-Fits Method

**Figure 10:**
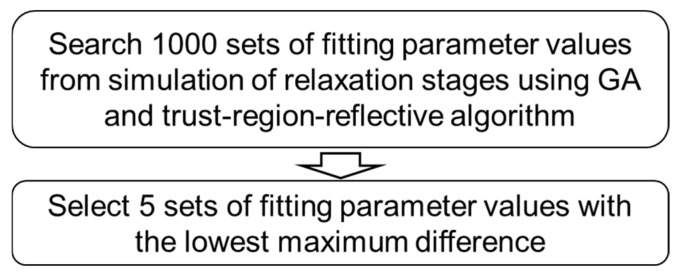
Procedures to determine the model parameter values using the best-five-fits method.

The best-five-fits method can also be found in our previous work. Figure 10 shows the algorithm for this method. Similarly, 1000 sets of fitting parameter values were first determined using the method shown in Figure 8. Then, the five sets of fitting parameter values with the lowest maximum difference between experimental data and simulation data were selected. Therefore, this method can provide model parameter values with high accuracy for the simulation, that is, the maximum difference between experimental data and simulation data is less than 0.08 MPa.

## 5. Results and Discussion

**Figure 11:**
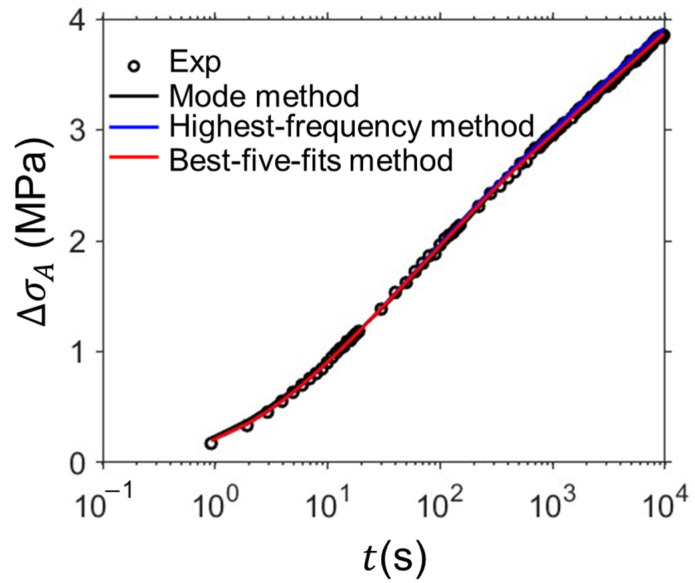
Sample curves of relaxation data (at 1.197 mm of stroke) from experiments (black circles), simulation from mode method (black line), highest-frequency method (blue line), and the best-five-fits method (red line).

**Figure 12:**
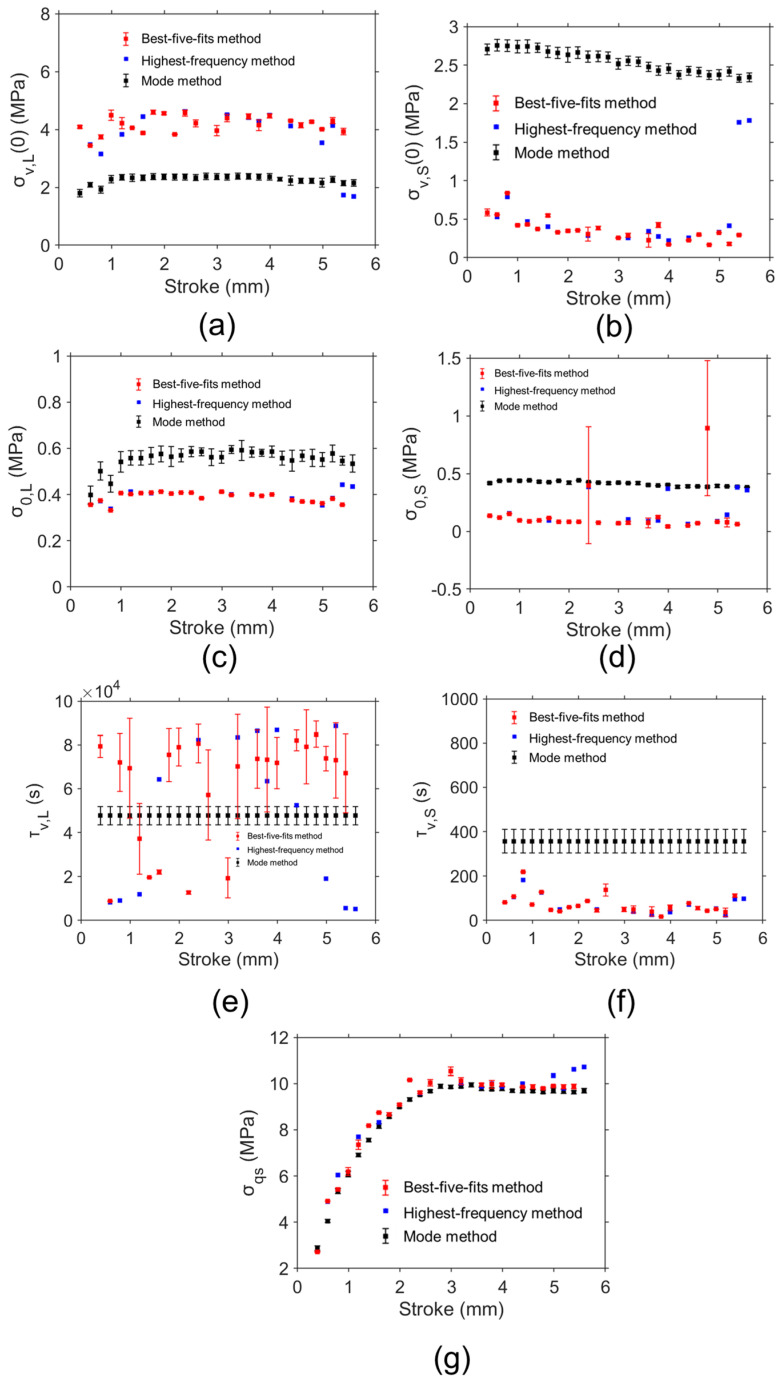
Model parameter values determined by different methods using the relaxation data from the RR test on HDPE cylindrical specimen: (**a**) $\sigma_{v,L}(0)$, (**b**) $\sigma_{v,S}(0)$, (**c**) $\sigma_{0,L}$, (**d**) $\sigma_{0,S}$, (**e**) $\tau_{v,L}$, (**f**) $\tau_{v,S}$, and (**g**) $\sigma_{qs}$.

The fitting parameter values at different deformation levels determined from the four methods were compared in this section. Figure 11 shows sample curves of relaxation data from experiments and simulations from the mode method, highest-frequency method, and the best-five-fits method, which indicates these methods are able to provide accurate simulations of the relaxation behavior. Figure 12 shows the model parameter values determined by the mode method, highest-frequency method, and the best-five-fits method based on the RR test data of HDPE cylindrical specimens. Ten sets of model parameter values were determined using the mode method for the evaluation of the variation in model parameter values. The red and black squares represent the average values of the model parameter values determined from the best-five-fits method and mode method, respectively. The error bar in the figure was used to show the standard deviation of the model parameter values. The blue squares show the results for model parameter values determined from the highest-frequency method. It is important to note that some blue squares were missed at several deformation levels. The reason is that the combination of the model parameter values with the highest frequency may not be the optimal set of model parameter values for accurate simulation of the experimental data. Therefore, the disadvantage of the highest-frequency method is the absence of model parameter values at several deformation levels.

Figure 12a shows that $\sigma_{v,L}(0)$ values determined from the best-five-fits method and highest-frequency method are close. Similarly consistent results from the best-five-fits method and highest-frequency method can also be found for $\sigma_{v,S}(0)$, $\sigma_{0,L}$, $\sigma_{0,S}$, $\tau_{v,L}$, and $\tau_{v,S}$ in Figure 12b, Figure 12c, Figure 12d, Figure 12e, and Figure 12f, respectively.

From Figure 12a, $\sigma_{v,L}(0)$ values from the best-five-fits method and highest-frequency method are much larger than those determined from the mode method at the same deformation level for most deformation levels. However, Figure 12b shows that $\sigma_{v,S}(0)$ values determined from the best-five-fits method and highest-frequency method are much smaller than those determined from the mode method at the same deformation level. From the best-five-fits method and highest-frequency method, $\sigma_{v,S}(0)$ values are lower than $\sigma_{v,L}(0)$ values at most deformation levels. $\sigma_{v,L}(0)$ values from the highest-frequency method fluctuate from 1 mm to 5 mm and show a decreasing trend after 5 mm. $\sigma_{v,S}(0)$ values show a decreasing trend after 1 mm.

From Figure 12c, $\sigma_{0,L}$ values from the best-five-fits method and highest-frequency method are much smaller than those determined from the mode method at the same deformation level, and a similar pattern can also be found for the $\sigma_{0,S}$ values in Figure 12d. $\sigma_{0,L}$ values from the highest-frequency method vary sharply from 0 mm to 1 mm and fluctuate after 1 mm. From the best-five-fits method and highest-frequency method, $\sigma_{0,S}$ values are lower than $\sigma_{0,L}$ values at most deformation levels. $\sigma_{0,S}$ values from the highest-frequency method are almost constant during deformation. From Figure 12e, $\tau_{v,L}$ values from the best-five-fits method and highest-frequency method are scattering during deformation. It was reported that that inaccurate values for the characteristic relaxation time have a minor influence on the simulation [28]. Therefore, the scattering $\tau_{v,L}$ values may be caused by their sensitivity in the simulation. Figure 12f shows $\tau_{v,S}$ values from the best-five-fits method and highest-frequency method are lower than those from the mode method. $\tau_{v,L}$ values and $\tau_{v,S}$ values from the highest-frequency method vary during deformation. Figure 12g indicates that the $\sigma_{qs}$ values from the mode method, best-five-fits method, and highest-frequency method are close. In addition, the standard deviation for $\sigma_{qs}$ values determined by the mode method and best-five-fits method is quite small.

**Figure 13:**
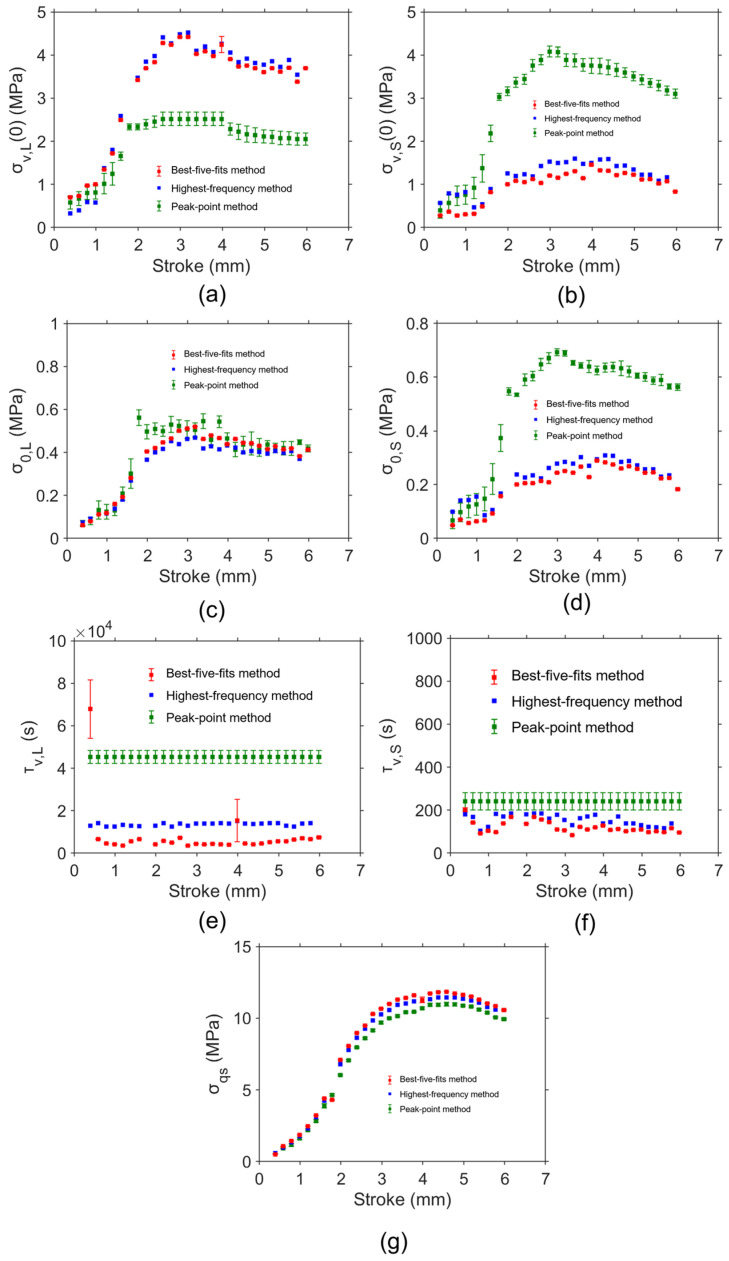
Model parameter values determined by different methods using the relaxation data from the RR test on PE-Xa NPR specimen: (**a**) $\sigma_{v,L}(0)$, (**b**) $\sigma_{v,S}(0)$, (**c**) $\sigma_{0,L}$, (**d**) $\sigma_{0,S}$, (**e**) $\tau_{v,L}$, (**f**) $\tau_{v,S}$, and (**g**) $\sigma_{qs}$.

Figure 13 shows the model parameter values determined by the peak-point method, highest-frequency method, and the best-five-fits method based on the RR test data for the PE-Xa NPR specimen. Ten sets of model parameter values were determined using the peak-point method for the evaluation of the variation in model parameter values. The red and green squares represent the average values of the model parameter values determined from the best-five-fits method and peak-point method, respectively. The error bar in the figure was used to show the standard deviation of the model parameter values. The blue squares show the results for model parameter values determined from the highest-frequency method.

Figure 13a shows that $\sigma_{v,L}(0)$ values determined from the best-five-fits method and highest-frequency method are close. Similarly consistent results from the best-five-fits method and highest-frequency method can also be found for $\sigma_{v,S}(0)$, $\sigma_{0,L}$, $\sigma_{0,S}$, $\tau_{v,L}$, and $\tau_{v,S}$ in Figure 13b, Figure 13c, Figure 13d, Figure 13e, and Figure 13f, respectively. This is consistent with the finding from the results for the HDPE cylindrical specimen. From the best-five-fits method and highest-frequency method, $\sigma_{v,S}(0)$ values are lower than $\sigma_{v,L}(0)$ values at most deformation levels. $\sigma_{v,L}(0)$ values and $\sigma_{v,S}(0)$ values determined from the highest-frequency method increase and then decrease with increasing deformation.

From Figure 13a, $\sigma_{v,L}(0)$ values from the best-five-fits method and highest-frequency method are much larger than those determined from the peak-point method at the same deformation level for most deformation levels. On the contrary, Figure 13b shows that $\sigma_{v,S}(0)$ values determined from the best-five-fits method and highest-frequency method are much smaller than those determined from the peak-point method at the same deformation level.

It is important to note that $\sigma_{0,L}$ values from the three methods are close in Figure 13c. In addition, the standard deviation of $\sigma_{0,L}$ values from the best-five-fits method and peak-point method is quite small, indicating good consistency. From Figure 13d, $\sigma_{0,S}$ values from the best-five-fits method and highest-frequency method are lower than those from the peak-point method for most deformation levels. From the best-five-fits method and highest-frequency method, $\sigma_{0,S}$ values are lower than $\sigma_{0,L}$ values at most deformation levels. $\sigma_{0,L}$ values and $\sigma_{0,S}$ values determined from the highest-frequency method increase and then decrease with increasing deformation.

From Figure 13e, $\tau_{v,L}$ values from the best-five-fits method and highest-frequency method are lower than those from the peak-point method at most deformation levels. A similar pattern was found for $\tau_{v,S}$ in Figure 13f. $\tau_{v,L}$ values and $\tau_{v,S}$ values from the highest-frequency method fluctuate during deformation.

From Figure 13g, $\sigma_{qs}$ values from the peak-point method, best-five-fits method, and highest-frequency method are close. In addition, the standard deviation for $\sigma_{qs}$ values determined by the peak-point method and best-five-fits method is relatively small.

Therefore, for the HDPE cylindrical specimen and PE-Xa NPR specimen, model parameter values from the best-five-fits method and highest-frequency method are close. $\sigma_{qs}$ values can be consistent for different parameter value identification methods. $\sigma_{qs}$ values for the HDPE cylindrical specimen and PE-Xa NPR specimen increase first and then reach a plateau. These two specimen types differ significantly in geometry, which inherently affects their stress distribution and relaxation behavior. As a result, the absolute values of the identified parameters are not directly comparable between the two specimen types. From the best-five-fits method and highest-frequency method, $\sigma_{v,S}(0)$ values are lower than $\sigma_{v,L}(0)$ values at most deformation levels, and $\sigma_{0,S}$ values are lower than $\sigma_{0,L}$ values at most deformation levels. As demonstrated in Figure 12 and Figure 13, the highest-frequency method fails to generate model parameter values at several deformation levels. The mode method and peak-point method contain assumptions during simulation. The maximum values for the standard deviation of $\sigma_{v,L}\left(0\right)\;values$, $\sigma_{v,S}(0)$ values, and $\sigma_{qs}$ values are all less than 0.5 MPa, and this value is much lower than the maximum applied stress. Therefore, the best-five-fits method is considered to provide the most reliable and relatively unique set of model parameter values, which is considered a practical approach to model parameter value identification for PE. In contrast, the mode method, peak-point method, and highest-frequency method are exploratory, optional, and comparative approaches. The two data points in Figure 12d and Figure 13e that deviate significantly from the overall trend may result from material defects or experimental noise, which can affect stress relaxation behavior at specific deformation levels.

From the literature [20], reference stress in the spring-dashpot model is related to the activation volume of PE. Figure 13c,d show that the $\sigma_{0,L}$ and $\sigma_{0,S}$ values increase with increasing deformation before 3 mm, which indicates the values of activation volume decrease with the increase in deformation. This trend is consistent with the findings in the literature [20,23]. According to Aliotta et al. [33], works in the literature on the correlation of the stress relaxation to activation volume are limited. From Gao et al. [34], the activation volume is the movement space of a single chain within a lamellar cluster from the initial state to the yield point. Xie et al. [35] proposed that a higher activation volume represents a larger lamellar cluster thickness and lower pore number in unit area. The molecular chain motivation and extensibility improves when the activation volume increases. Strobl’s group [20] considered that the quasi-static stress is contributed by the amorphous network and crystal skeleton. In our previous work [27], it was found that the long-term branch generates positive stress and the short-term branch generates negative stress in the recovery behavior of PE. It was reported that the negative stress generated by the short-term branch should represent the stress response from the amorphous phase [36,37,38]. A study to correlate the model parameters and the microstructures was being planned when the manuscript was prepared. In addition, each relaxation stage of the RR test is treated independently, and as such, the model cannot fully account for prior strain rates or load history effects currently. A further study is being planed to find the history dependence of the spring-dashpot model.

## 6. Conclusions

This paper presents four parameter value identification methods (the mode method, best-five-fits method, peak-point method, and highest-frequency method) for the relaxation behavior from RR tests on cylindrical and NPR specimens, respectively. Results from the RR tests can be accurately mimicked using the three-branch model with parameter values determined using four proposed methods, and the values for the maximum difference between the stress measured experimentally and those determined from the model using the mode method, best-five-fits method, peak-point method, and highest-frequency method are less than 0.08 MPa, 0.08 MPa, 0.1 MPa, and 0.08 MPa, respectively. These values are much smaller than the values reported in the literature, such as 0.17 MPa [30], 0.3 MPa [31], 0.4 MPa [23], and 1 MPa [32]. The model parameter values determined by the four methods were compared. $\sigma_{qs}$ values can be consistent for different parameter value identification methods. From the best-five-fits method and highest-frequency method, $\sigma_{v,S}(0)$ values were lower than $\sigma_{v,L}(0)$ values after 1 mm for the NPR specimen, and $\sigma_{0,S}$ values were lower than $\sigma_{0,L}$ values after 1 mm for the NPR specimen. The best-five-fits method has been demonstrated to provide the most reliable and relatively unique set of model parameter values, making it a practical approach to model parameter value identification for PE. In contrast, the mode method, peak-point method, and highest-frequency method are exploratory, optional, and comparative approaches. The four proposed methods provide valuable insights into the challenges of parameter identification for nonlinear spring-dashpot models. This also contributes to the determination of time-independent, quasi-static stress and time-dependent, viscous stress in SCPs.

## Data Availability

The data presented in this study are available on request from the corresponding author. The data are not publicly available due to privacy restrictions.

## Supplementary Materials

The following supporting information can be downloaded at: https://www.mdpi.com/article/10.3390/ma18132960/s1. Table S1: The ranges for the boundaries shown in the Figure 4; Table S2: The ranges for the boundaries shown in the Figure 6.
