# Supplementary material for: Evaluating Parameter Value Identification Methods for Modeling of Nonlinear Stress Relaxation in Polyethylene
Source: Materials (Basel). 2025 Jun 23;18(13):2960. doi: 10.3390/ma18132960 (PMC12250635; doi:10.3390/ma18132960)
Supplement: Supplementary file 1 [file materials-18-02960-s001.zip › materials-3641691-supplementary.pdf]

The ranges of the boundaries used in the Figure 4 are shown in the Table S1.

Table S1. The ranges for the boundaries shown in the Figure 4.

Boundaries	Ranges
(1)	➢ $\sigma_{v,L}(0)$: [0.1, 20]<br>➢ $\sigma_{0,L,}$: [$\sigma_{0,ci} - 0.2$, $\sigma_{0,ci} + 0.2$]<br>➢ $\tau_{v,L}$: [1000, 90000]<br>➢ $\sigma_{v,S}(0)$: [0.1, 20]<br>➢ $\sigma_{0,S}$: [0.01, 2]<br>➢ $\tau_{v,S}$: [1, 900]
(2)	➢ $\tau_{v,L}$: [avg($\tau_{v,L,i,k-1}$) $- 10^{5-k}$, avg($\tau_{v,L,i,k-1}$) $+ 10^{5-k}$]<br>➢ $\tau_{v,S}$: [avg($\tau_{v,S,i,k-1}$) $- 10^{3-k}$, avg($\tau_{v,S,i,k-1}$) $+ 10^{3-k}$]
(3)	➢ $\tau_{v,L}$: [min($\tau_{v,L,i}$), max($\tau_{v,L,i}$)]<br>➢ $\sigma_{0,S}$: [min($\sigma_{0,S,i}$), max($\sigma_{0,S,i}$)]<br>➢ $\tau_{v,S}$: [min($\tau_{v,S,i}$), max($\tau_{v,S,i}$)]
(4)	➢ $\sigma_{v,L}(0)$: [a, b]
(5)	➢ $\sigma_{v,L}(0)$: [0.1, b] ($2 \leq i \leq 30$)
(6)	➢ $\sigma_{v,L}(0)$: [0.1, $\sigma_{v,L}(0)$ in relaxation i]<br>➢ $\sigma_{0,L}$: [0.01, $\sigma_{0,L}$ in relaxation $i+1$]<br>➢ $\tau_{v,L}$: [γ, φ] (variation is less than 1 s)<br>➢ $\sigma_{v,S}(0)$: [− 20, − 0.01]<br>➢ $\sigma_{0,S}$: [0.01, $\sigma_{0,S}$ in relaxation $i+1$]<br>➢ $\tau_{v,S}$: [1, 10000]
(7)	➢ $\tau_{v,S}$: [avg($\tau_{v,S,i,(k-1)}$) $- 10^{4-k}$, avg($\tau_{v,S,i,(k-1)}$) $+ 10^{4-k}$]
(8)	➢ $\sigma_{0,S}$: [a, b]<br>➢ $\tau_{v,S}$: [min($\tau_{v,S,i}$), max($\tau_{v,S,i}$)]
(9)	➢ $\sigma_{v,S}(0)$: [c, d]
(10)	➢ $\sigma_{v,L}(0)$: [$y_i - \sigma_{v,S,i}(0) - 0.04$, $y_i - \sigma_{v,S,i}(0) + 0.04$]<br>➢ $\sigma_{v,S}(0)$: [$\sigma_{v,S,i}(0) - 0.04$, $\sigma_{v,S,i}(0) + 0.04$]
(11)	➢ $\sigma_{v,L}(0)$: [$y_i - \sigma_{v,S,i}(0) - 0.04$, $y_i - \sigma_{v,S,i}(0) + 0.04$]<br>➢ $\sigma_{0,L}$: [p, q]<br>➢ $\sigma_{v,S}(0)$: [$\sigma_{v,S,i}(0) - 0.04$, $\sigma_{v,S,i}(0) + 0.04$]

The ranges of the boundaries used in the Figure 6 are shown in the Table S2.

Table S2. The ranges for the boundaries shown in the Figure 6.

Boundaries	Ranges
(1)	➢ $\sigma_{v,L}(0)$: [0.1, 20]<br>➢ $\sigma_{0,L,}$: [0.5$\sigma_{0,ci}$,1.5$\sigma_{0,ci}$]<br>➢ $\tau_{v,L}$: [1000, 90000]<br>➢ $\sigma_{v,S}(0)$: [0.1, 20]<br>➢ $\sigma_{0,S}$: [0.01, 2]<br>➢ $\tau_{v,S}$: [1, 900]
(2)	➢ $\tau_{v,L}$: [avg($\tau_{v,L,i,k-1}$) $-\ 10^{5-k}$, avg($\tau_{v,L,i,k-1}$) $+\ 10^{5-k}$]<br>➢ $\tau_{v,S}$: [avg($\tau_{v,S,i,k-1}$) $-\ 10^{3-k}$, avg($\tau_{v,S,i,k-1}$) $+\ 10^{3-k}$]
(3)	➢ $\tau_{v,L}$: [min($\tau_{v,L,i}$), max($\tau_{v,L,i}$)]<br>➢ $\tau_{v,S}$: [min($\tau_{v,S,i}$), max($\tau_{v,S,i}$)]
(4)	➢ $\tau_{v,L}$: [min($\tau_{v,L,i}$), max($\tau_{v,L,i}$)]<br>➢ $\tau_{v,S}$: [min($\tau_{v,S,i}$), max($\tau_{v,S,i}$)]<br>➢ $\sigma_{v,L}(0)$: [$\sigma_{v,L,i-1}(0)$, $\sigma_{v,L,m}(0)$]
(5)	➢ $\tau_{v,L}$: [min($\tau_{v,L,i}$), max($\tau_{v,L,i}$)]<br>➢ $\tau_{v,S}$: [min($\tau_{v,S,i}$), max($\tau_{v,S,i}$)]<br>➢ $\sigma_{v,L}(0)$: [0.1, $\sigma_{v,L,i-1}(0)$]
